# The Hollow Adrenal Gland Sign: An Ominous Alert

**DOI:** 10.5334/jbsr.2830

**Published:** 2022-06-28

**Authors:** Ana Costa, Marta Baptista

**Affiliations:** 1 Hospital Prof. Doutor Fernando Fonseca, E.P.E., Amadora, PT

**Keywords:** adrenal gland, septic shock

## Abstract

**Teaching Point:** The hollow adrenal gland sign is common, and may be specific, in patients with septic shock, and is a predictor of poor prognosis.

## Case Report

**Figure 1:**
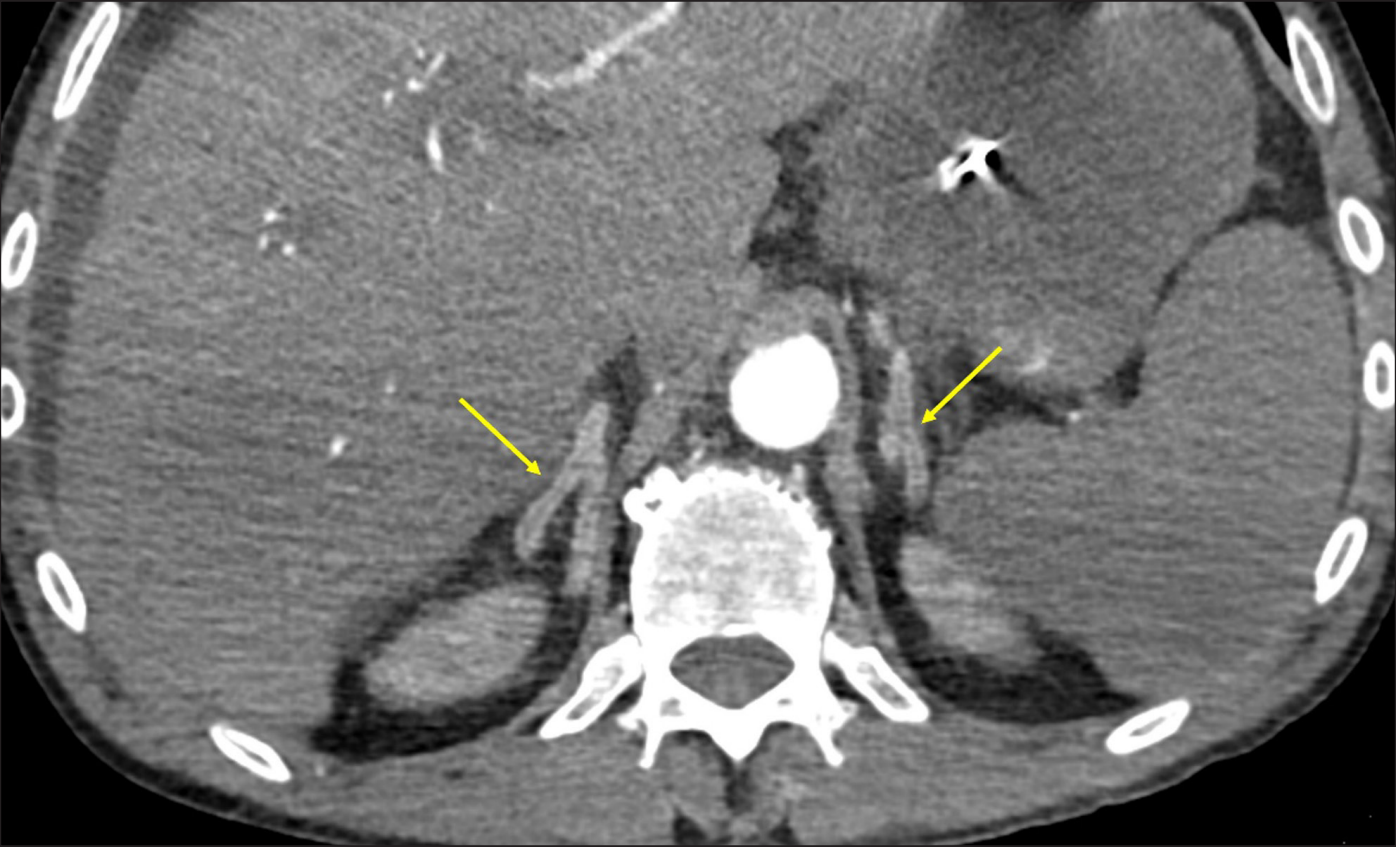


**Figure 2:**
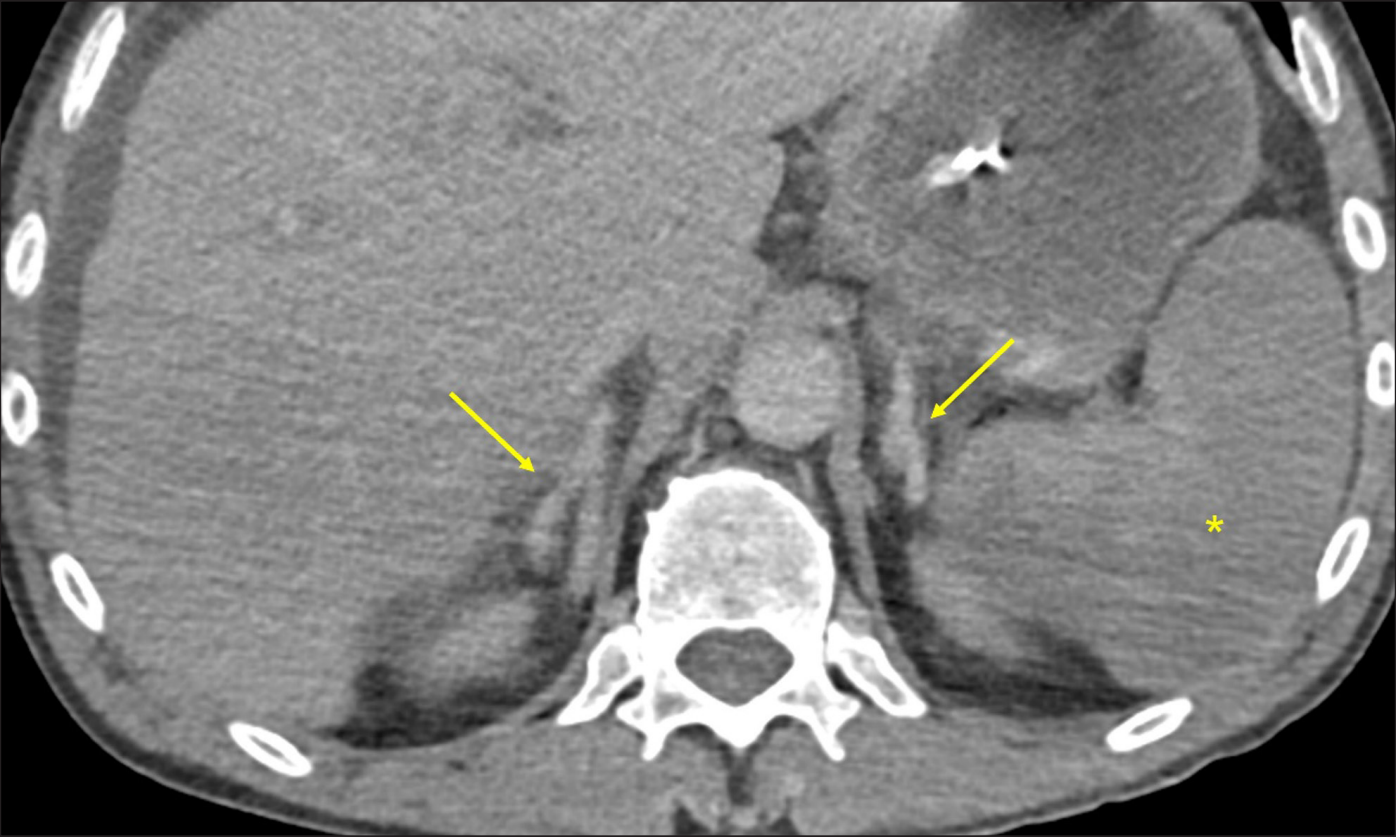


**Figure 3:**
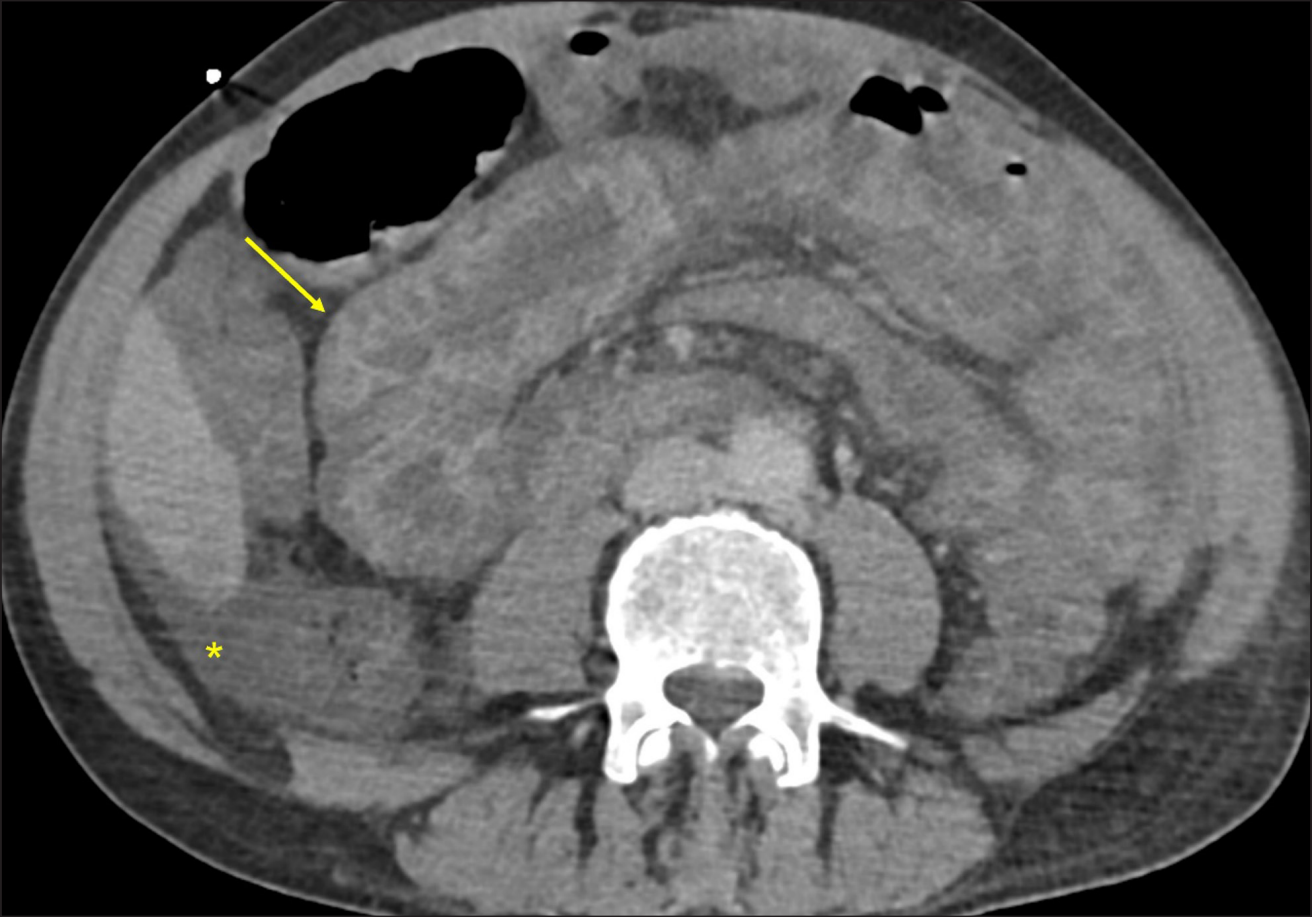


A 58-year-old male, smoker, with a history of alcohol abuse presented to the emergency department due to fever (38.2°C). The patient had bilateral scleral icterus, hepatomegaly, and bilateral lower limb edema. Laboratory tests revealed elevation of inflammatory parameters, thrombocytopenia, acute kidney injury, hyperbilirubinemia, cytocholestasis, and coagulopathy. An acute-on-chronic liver failure syndrome due to an infection of unknown origin was presumed. His condition deteriorated with septic shock and abdominal pain. Nasogastric entubation revealed faecaloid content. He underwent an abdominopelvic computed tomography with intravenous contrast (80ml 300mg iodine/mL at 4mL/s, followed by a 50mL saline flush) on arterial and venous phases. Adrenal glands showed central hypoenhancement relative to the peripheral zone in the arterial phase (arrows in Figure 1) and homogenous enhancement in the venous phase (arrows in Figure 2) – the hollow adrenal gland sign (HAGS). Images also demonstrated ascites (asterisk in Figure 3), thickened bowel loops due to submucosal edema and mucosal hyperenhancement involving the small bowel (arrow in Figure 3) and the right colon, splenic hypoenhancement (asterisk in Figure 2), and some bilateral renal infarcts (not shown). These findings were interpreted as a hypoperfusion complex syndrome. There were also signs of segmental acute intestinal ischemia (not shown), so the patient underwent an emergent laparotomy. There was bad perfusion of the small bowel and right colon, with no signs of irreversible ischaemia, so a peritoneal lavage was performed. Subsequent fluorescence angiography confirmed bowel viability. Unfortunately, the patient died.

## Comment

The HAGS is a pattern recently described in the literature on dual phase contrast-enhanced computed tomography (CT) in patients with septic shock [1]. It is defined as a less intense enhancement of the central zone of the adrenal gland in the arterial phase, followed by a homogeneously enhancing adrenal gland in the venous phase. There are two types: the hypoenhancement of the central zone is homogeneous, forming a typical Y or I-shaped region; or the central zone has a heterogeneous enhancement creating a mosaic appearance. The sign is present when it appears on either adrenal gland. The HAGS is common, and may be specific, in patients with septic shock. It is associated with severe illness more frequently requiring organ-supportive interventions and it has a good specificity to predict poor prognosis; however, it has a low sensitivity. The HAGS showed excellent reproducibility between different observers, suggesting it is easily recognizable and can be used to identify patients with sepsis who have a higher risk of in-hospital mortality and select the appropriate level of care. A similar but rarer enhancement pattern, the “train-track appearance,” is defined as preservation of normal peripheral adrenal enhancement with central low attenuation in a thickened adrenal [2]. In this cases, auxiliary features were described, namely peri-adrenal fat stranding. This has been reported in early non-traumatic adrenal hemorrhage, which is an uncommon but potentially life-threatening condition, preceding adrenal hematomas.
